# Engineering of a rough auxotrophic mutant *Salmonella* Typhimurium for effective delivery

**DOI:** 10.18632/oncotarget.25192

**Published:** 2018-05-22

**Authors:** Jonathan Lalsiamthara, Je Hyoung Kim, John Hwa Lee

**Affiliations:** ^1^ College of Veterinary Medicine, Chonbuk National University, Iksan Campus, Iksan 54596, Republic of Korea

**Keywords:** Salmonella Typhimurium, live-attenuated bacterial vector, pre-existing immunity, heterologous antigen delivery

## Abstract

Live *Salmonella* vaccine vectors offer a remarkable platform for delivering immunogens and therapeutic molecules by mimicking natural intracellular infections; however, pre-existing anti-vector immunity can impede effective deployment. Measures to alleviate pre-existing immunity include the use of heterologous vectors, development of highly attenuated strain enabling greater payload, removal of major immunoreactive components from the vector, and/or augmentation of delivered antigens via increased presentation in antigen presenting cells. Here we report a *Salmonella* Typhimurium (ST) vector-JOL1800 that embodies these requisite properties. JOL1800 is a highly attenuated, auxotrophic, and O-antigen deficient rough-mutant strain. Heterologous bacterial and viral antigens were expressed and delivered using JOL1800 in mice, irrespective of the inoculation route successful inductions of the mucosal and systemic humoral responses were observed. Compared to smooth LPS vector delivery, we observed an increased fraction of delivered-antigen presenting dendritic cells and a higher frequency of delivered-antigen displayed per macrophage. Upon post-priming with JOL1800 delivery, efficacy of the delivery was minimally affected as indicated by insignificant decrease in colonization, humoral and cellular responses. Our results show that the generated vector is capable of remote antigen delivery, manifests higher antigen presentation, is Differentiating Infected from Vaccinated Animals (DIVA) capable, evades normal pre-existing immunity, and can be deployed for effective delivery.

## INTRODUCTION

*Salmonella enterica* serovar Typhimurium (ST) is a gram-negative intracellular pathogen and is one of the most extensively studied organisms particularly in the areas of basic science research, systemic bacterial infections, immunological profiling, and host resistance to pathogens. It is also widely studied for its use as a vaccine and live vector vaccine and also as an anti-tumor vector due to its intrinsic properties [1, 2]. With advancements in genetic engineering and synthetic biology, modified ST are designed to deliver a diverse range of payloads such as immunogens, heterologous antigens, therapeutic, and anti-tumor drug molecules [3]. The intracellular localization of ST allows for the cytosolic delivery of drugs and cytotoxic proteins that are otherwise unable to enter eukaryotic cells. ST is a facultative pathogen that is found within a variety of phagocytic and non-phagocytic cells [4]. Hence, a live ST vector that constitutively expresses beneficial proteins would deliver the antigen to various tissues of the host including systemic as well as mucosal sites.

Characteristics of an ideal bacterial delivery vector include sufficient attenuation, active antigen-presenting cell recognition, and stable maintenance of antigen coding-recombinant plasmid vector. In order to achieve attenuation, ST mutants deficient in the biosynthesis of metabolic precursors, energy carriers, or mutants defective in the global regulatory system have been the most widely characterized [5–7]. These mutants are excellent carriers for vaccine antigens from other bacteria [8, 9], viruses [10, 11], parasites [12–14], and tumors [15, 16] and are able to stimulate strong systemic and local immune responses against the corresponding antigens [17]. In this study, an attenuated mutant ST strain that was devoid of virulence-associated bacterial regulatory genes *lon* and *cpxR* [18, 19], was attenuated further via deletion of a gene that is involved in LPS biosynthesis pathway. It is known that deficiency in LPS resulted in increased sensitivity to complement mediated killings and renders the rough bacterial strains avirulent [20]. Rough *Salmonella* have been evaluated and tested extensively as a vaccine candidate [21–23]; however, to date, very few rough strains have been tested as a live vector vaccine [24]. Recently, our group has extensively employed smooth or rough *Salmonella* vectors for delivering mucosal adjuvants and *Brucella* antigens, and their application as vaccine candidates [25–27]. It has been demonstrated that O-antigen (O-Ag) of the *Salmonella* lipopolysaccharide (LPS) as an important factor for controlling the intracellular fate of *Salmonella* in dendritic cells (DC). A *Salmonella* strain without O-Ag showed an increased rate of uptake by DC and altered intracellular processing and increased degradation, which boosted the DC immune function [28]. Stable host-plasmid maintenance was attained via an antibiotic free, balanced-lethal host-vector system. An auxotrophic *asd* negative ST strain was complemented with plasmid-encoded aspartate semialdehyde dehydrogenase [27].

Despite potential and promise, the biggest challenge in implementing successful delivery depends in large part on the presence or absence of pre-existing immunity. Pre-existing anti-*Salmonella* immunity can impede repetitive use of the vector for the delivery of immunogens and therapeutic molecules [29, 30]. Measures that can alleviate these limitations include switching the ST serotype on subsequent deliveries or boosting at an appropriate time [29], administration of higher vector vaccine doses [31], and exposure of the host to the candidate antigen prior to vector-priming [32]. However, with ST-based vectors, pre-existing immunity may not be completely avoidable mainly due to the ubiquitous presence of *Salmonella*e in food and in the water system and also due to its endemic propensity in livestock and the human population [33]. Based on our hypothesis, an alternative approach to improve the ST vector would involve development of a highly attenuated strain that is safe even at high doses and thus yields higher payload delivery, removal of major bacterial surface immunoreactive components such as LPS [34] from the vector, and augmentation of delivered antigens via increased presentation in antigen-presenting cells.

Finally, removal of a portion of *Salmonella* LPS renders the organism insensitive to anti-LPS antibodies. Most surveillance agencies for food safety and *Salmonella* programs use LPS-based ELISA as detection [35]. Hence, a delivery system lacking a complete LPS would not interfere with *Salmonella* diagnostic programs. Hence, the added advantage of using a rough ST strain as a live vector vaccine include Differentiation of Infected and Vaccinated Animals (DIVA) capability, safer attenuated phenotypic profile, circumvention of pre-existing antibodies, and a broad range of cross protection.

Considering all of the practical advantages, we engineered a rough ST live bacterial vector vaccine, designated as JOL1800. We report here the development and characterization of an *rfaL* gene knock out O-Ag deficient mutant strain. The *rfaL* gene encodes for O-antigen ligase that adds the O-antigen on the glucose (II) group of LPS. To investigate the antigen delivery capabilities of the developed strain in this study, heterologous bacterial and viral antigens were expressed and delivered using JOL1800 through mucosal and systemic routes in mice. Antigen specific humoral responses were studied, including examining the presentation of delivered-antigens in DC and intraperitoneal macrophages, the effect of pre-existing anti-ST wild type immunity on delivery, and DIVA ability of the strain based on LPS indirect ELISA.

## RESULTS

### Construction and validation of JOL1800

**Figure 1:**
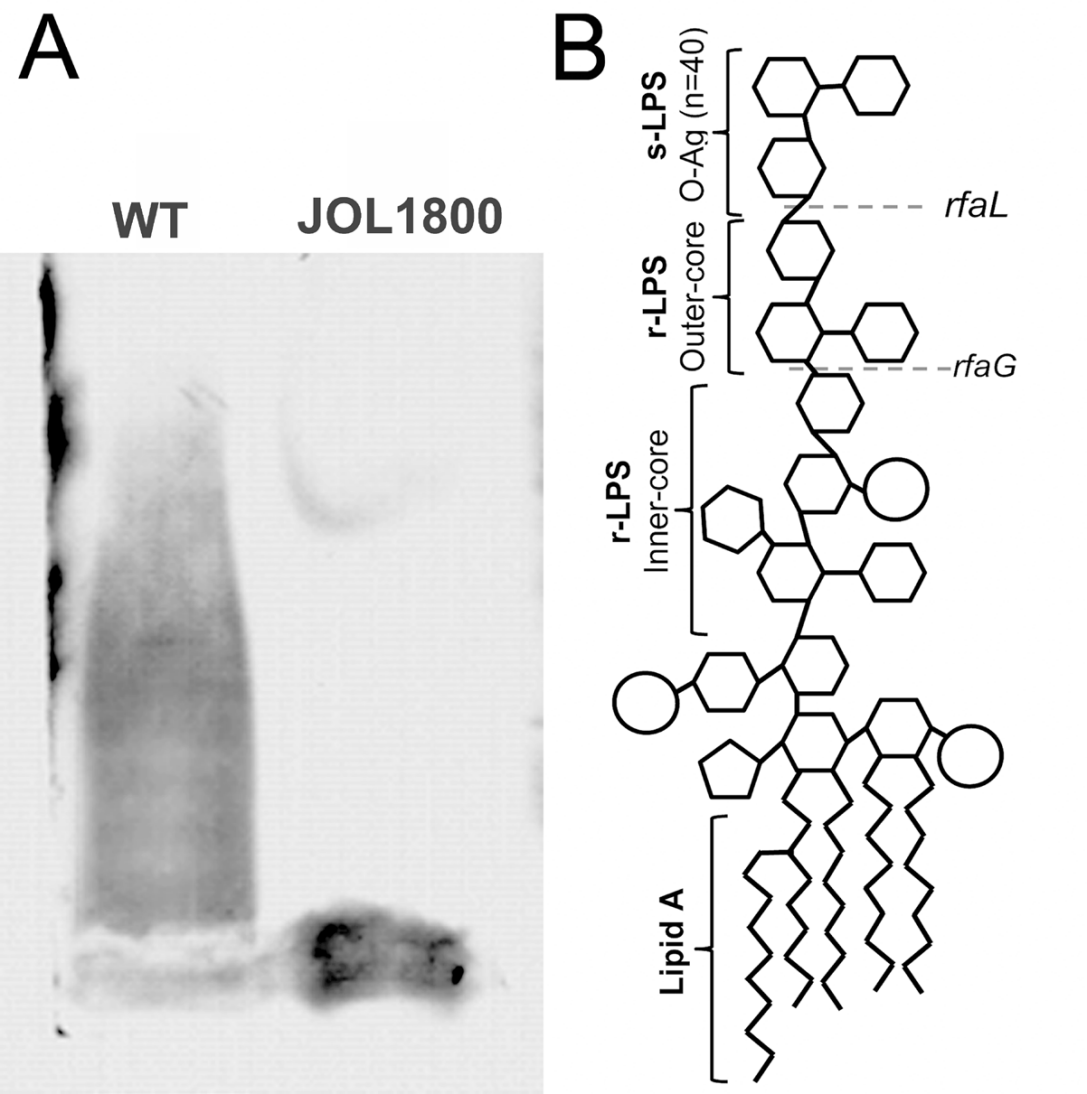
*Salmonella* LPS smooth and rough LPS from wild type ST and rough ST JOL1800 strains was extracted suing phenol based technique. Purified LPS was subjected to 15% SDS-PAGE electrophoresis. Separated LPS was stained with silver stain after periodic acid oxidation. (**A**) Lane ‘WT’ depicts wild type LPS with complete O-antigen. Lane ‘JOL1800’ depicts rough type LPS without O-antigen. (**B**) Schematic representation of *Salmonella* LPS (Adapted and modified for Kong *et al.* 2011 [24]). Dotted lines represent units affected by rfaL gene deletion.

In order to combine the properties of an ideal live vaccine vector, JOL1800 was engineered based on the highly immunogenic and invasive parental smooth ST strain JOL912 [36] (Table 1). JOL912 (Δ*lon*Δ*cpxR*Δ*asd*) is a triple gene knockout auxotrophic mutant that requires complementation of the *asd* gene to replicate. Dependence on the *asd*+ plasmid vector creates a balanced-lethal complementation between the host and the plasmid construct. A non-reverting *rfaL* deletion mutant was constructed using the red lambda recombineering approach. The O-Ag ligase gene *rfaL* was deleted from the JOL912 genome to generate the Δ*lon*Δ*cpxR*Δ*asd*Δ*rfaL* knockout mutant JOL1800. The deletion event was confirmed by using outer and inner PCR primers that detect the flanking regions of the target gene. Upon replacement of wild type *rfaL* with a *cat*^*R*^ gene cassette (∼1.1 kb), the original amplicon size of 1.9 kb was reduced to 1.7 kb in the mutant type. PCR primers designed to amplify the internal *rfaL* sequence yielded no amplification in the mutant strain (Supplementary Figure 1). Silver stains of PAGE-separated purified LPS extracts from JOL1800 revealed the absence of the major O-Ag (Figure 1A), while the wild type strains showed the complete LPS pattern. To ascertain bacterial morphology and surface integrity, JOL1800 was subjected to electron microscopy analysis. Despite multiple genes affecting membrane synthesis (i.e., *asd*, *rfaL* genes) and disruption of flagella-assembly, weakened or disrupted membrane structures were not evident from direct EM observation (Supplementary Figure 2A).

**Table 1:** Bacterial strains, plasmids and primers used in this study

Strain/plasmid/ primers	Description	Reference
JOL401	*Salmonella* Typhimurium wild type, SPI-1 *invAE*^+^ *hilA*^+^ *avr*^+^; SPI-2, amino acid permease; SPI-3, *mgtC*^+^; SPI4, ABC transporter; SPI5, *pipB*^+^	[53]
JOL912	JOL401 ∆*lon*, ∆*cpxR*, Δ*asd*; smooth ST strain	[36]
JOL1800	JOL912 *∆rfaL,* O-antigen deficient strain, improved bacterial delivery vector; rough ST strain	This study
JOL912L	JOL912 delivering heterologous bacterial antigen-autotransporter protein A (LatA) via pJHL65-L	Lab stock
JOL401H	JOL401 delivering viral antigen-hemagglutinin (HA) via pJHL65-H	Lab stock
JOL912H	JOL912 delivering viral antigen-hemagglutinin (HA) via pJHL65-H	Lab stock
JOL912B	JOL912 delivering heterologous bacterial antigen-*Brucella* lumazine synthase (BLS) via pJHL65-B	[25]
JOL1800L	JOL1800 delivering heterologous bacterial antigen-autotransporter protein A (LatA) via pJHL65-L	This study
JOL1800H	JOL1800 delivering viral antigen-hemagglutinin (HA) via pJHL65-H	This study
JOL1800B	JOL1800 delivering heterologous bacterial antigen-*Brucella* lumazine synthase (BLS) via pJHL65-B	[25]
Plasmids		
pKD46	oriR101-repA101ts; encodes lambda red genes (*exo, bet, gam*); native terminator (tL3); arabinose-inducible promoter for expression (ParaB); *bla*	[52]
pKD3	oriR6Kgamma, *bla* (amp^R^), *rgnB* (Ter), cat^R^, FRT	[52]
pCP20	helper plasmid, contains a temperature-inducible *flp* gene for removing the chloramphenicol resistance gene	[52]
pJHL65	*asd* + vector, pBR ori, β-lactamase signal sequence-based periplasmic secretion plasmid, 6xHis, high copy number plasmid	[58]
pJHL65-L	pJHL65 harbouring *Lawsonia intracellularis latA* gene (*Lawsonia* auto-transporter protein A) constitutively express under Ptrc promoter, secreted under *bla* secretory system	Lab stock
pJHL65-H	pJHL65 harbouring HA gene (low pathogenic avian influenza), constitutively express under Ptrc promoter, secreted under *bla* secretory system	Lab stock
pJHL65-B	pJHL65 harbouring *Brucella abortus* (BLS) gene, constitutively express under Ptrc promoter, secreted under *bla* secretory system	[25]
Primers		
rfaL P1	5′-tgtctcatcccaaacctattgtggagaaaagatgctaaccGTGTAGGCTGGAGCTGCTTC	This study
rfaL P2	5′-atgatggaaaacgcgctgataccgtaataagtatcagcgcATGGGAATTAGCCATGGTCC	
rfaL DEL OT F	5′-GGATACGATAAACCGCAGTCG	This study
rfaL DEL OT R	5′- AACCGTGCGCTTGCTGATAAG	
rfaL DEL IN F	5′- ACAAGTTTAGGACTTCGCTGCC	This study
rfaL DEL IN R	5′-CAGAATGGTATTATGCGGACCG	
TYPH-F	5′-TTGTTCACTTTTTACCCCTGAA	[54]
TYPH-R	5′-CCCTGACAGCCGTTAGATATT	

### Removal of O-Ag from JOL1800 increased sensitivity to serum complement

**Figure 2:**
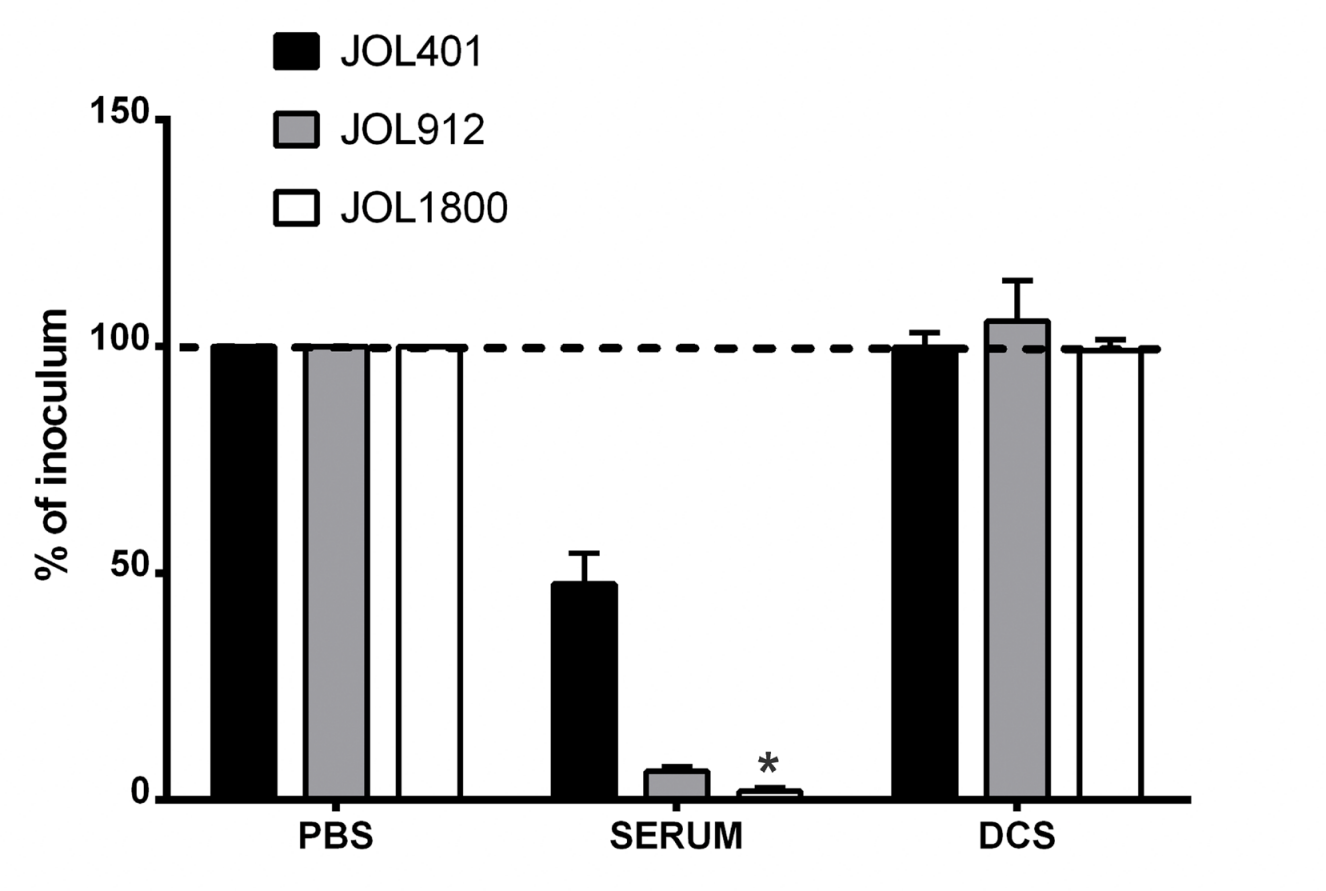
Sensitivity to complement killing *Salmonella* strains were evaluated for sensitivity to serum complement. Bacterial strains namely, wild type strain (JOL401), smooth strain (JOL912) and rough strain (JOL1800) were incubated with 50% fresh rabbit complement for 1 hr at 37° C. The degree of reduction due to complement activity was ascertained following plating on BGA plates. The rough strain JOL1800 showed increase sensitivity to complement killing. DCS- de-complemented serum. ^*^indicates significant difference (*P* ≤ 0.05) as compared to corresponding wild type control.

To characterize the phenotype of JOL1800 and to verify that the mutation resulted in a surface phenomenon, *in vitro* complement sensitivity assays were performed. We also investigated the effects of the complement to mediate bacterial cell lysis on the shortened O-Ag side chain strain. The JOL1800 rough strain was more sensitive to rabbit complement death than were the smooth strain counterparts (Figure 2). A significant reduction in bacterial count was observed compared to the wild type smooth strain JOL401 and parental smooth strain JOL912. In addition, we observed an increased sedimentation rate of the JOL1800 broth culture (Supplementary Figure 2B) and a marked acriflavine dye-agglutination of JOL1800 colonies (Supplementary Figure 2B). Rough strains of *Salmonella* have increased surface hydrophobicity upon removal of O-Ag, which increases auto-agglutination of the bacterial cells and thus imparts mass formation and quicker sedimentation.

### Attenuated JOL1800 persists long enough in the murine spleens to induce an effective immune response

Multiple gene mutations tend to yield extremely attenuated progeny strains, and some rough strains fail to reach target organs and fail to effectively colonize. Balanced attenuation is essential, where the ST strains should neither reside too long in the host, leading to chronicity, nor too short, resulting in failure to mount robust immunity. To ascertain the persistence of JOL1800 in the host system, splenic bacterial clearance interval was determined. Spleens of JOL-strain infected mice were homogenized in PBS, and the homogenate was directly plated; simultaneously, the remaining homogenate was subjected to further selective enrichment. All JOL401, JOL912, and JOL1800 strains were detected in the spleens of mice at 3, 7, and 14 days post inoculation, with varying levels of bacterial load (Table 2). JOL401 and JOL912 were detected beyond 14 days post inoculation. The recovery time (RT_50_) of JOL401, JOL912, JOL1800-oral, and JOL1800-IM were determined as 25.0, 15.8, 6.0, and 5.2 days, respectively (Supplementary Figure 3), indicating that JOL1800 is more attenuated than the other strains but also sufficiently resides in the host system long enough to efficiently deliver antigen and induce the host immune response. Splenomegaly was observed in JOL401-infected mice, while milder splenic enlargements were observed in mice infected with JOL912 or JOL1800 (Supplementary Figure 4).

**Table 2:** Splenic bacterial recovery of ST strains

Strain	3^a^		7		14		21	
	Log_10_	No. of positive	Log_10_	No. of positive	Log_10_	No. of positive	Log_10_	No. of positive
JOL401	3.06 ± 0.08^b^	8/8^c^ (8)^d^	2.67 ± 0.09	8/8 (8)	2.46 ± 0.16	8/8 (7)	2.03 ± 0.18	7/8 (5)
JOL912	1.26 ± 0.28^*^	8/8 (7)	1.93 ± 0.47	7/8 (6)	0.67 ± 0.38^*^	6/8 (5)	0.47 ± 0.23^*^	2/8 (0)
JOL1800 ORAL	0.64 ± 0.36^*^	8/8 (8)	1.59 ± 0.15^*^	6/8 (4)	0.30 ± 0.17^*^	4/8 (3)	0.0^*^	0/8 (0)
JOL1800 IM	0.73 ± 0.19^*^	8/8 (7)	1.74 ± 0.14^*^	7/8 (5)	0.10 ± 0.10^*^	3/8 (2)	0.0^*^	0/8 (0)

^a^days post inoculation.

^b^mean log10 splenic count ± standard error of mean.

^c^ total number of mice positive of ST strain by total number of mice screened.

^d^number of ST positive mice detected by direct BGA plating, without RV broth enrichment.

Asterisk ^*^indicates significant difference (*P* ≤ 0.05) as compared to corresponding wild type strain JOL401 for each corresponding day post inoculation.

### Live ST vector can remotely activate systemic and mucosal immunity by delivering immunogens to afflicted sites, irrespective of immunization site

**Figure 3:**
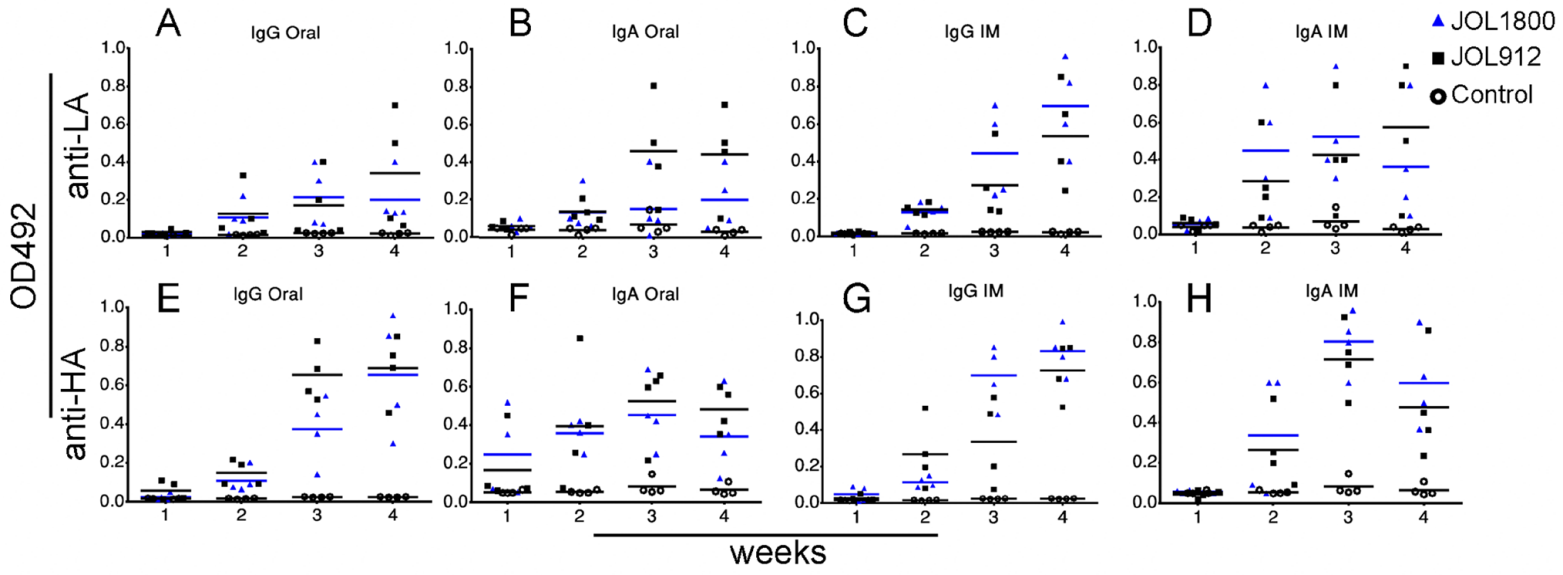
Humoral responses to delivered antigen The capability and versatility of *Salmonella* vectors for delivering diverse bacterial/viral antigens and subsequent induction of serum IgG and intestinal IgA humoral responses were evaluated using bacterial antigen. (**A**) IgG response of mice groups immunized with smooth JOL912L and rough JOL1800L delivery strains delivering LA antigen via oral route. (**B**) IgA response of mice groups immunized with smooth JOL912L and rough JOL1800L delivery strains delivering LA antigen via oral route. (**C**) IgG response of mice groups immunized with JOL delivery strains delivering LA antigen via IM route. (**D**) IgA response of mice groups immunized with JOL delivery strains delivering LA antigen via IM route. (**E**) IgG response of mice groups immunized with smooth JOL912H and rough JOL1800H delivery strains delivering HA antigen via oral route. (**F**) IgA response of mice groups immunized with smooth JOL912H and rough JOL1800H delivery strains delivering HA antigen via oral route. (**G**) IgG response of mice groups immunized with JOL delivery strains delivering HA antigen via IM route. (**H**) IgA response of mice groups immunized with JOL delivery strains delivering HA antigen via IM route. Blue bar represent mean OD492, colored bars are used to help differentiate the groups.

The versatility of JOL1800 in delivering diverse heterologous bacterial/viral antigens and thereby elicit the humoral immune response was investigated. Mice were inoculated with JOL912 and JOL1800 strains constitutively expressing the bacterial and viral antigens, *Lawsonia* auto-transporter protein A (LA) and hemagglutinin from H1N1 (HA), respectively. Murine IgG and SIgA antibody production against the specific antigens was determined using indirect ELISA (Figure 3). Further, to validate the hypothesis that live ST delivery can evoke humoral responses against delivered antigens remotely, anti-LA and anti-HA antibodies were determined from murine intestinal lavage samples after IM (systemic) immunization, and vice versa, for systemic serum IgG from mice immunized via the oral (mucosal) route. Interestingly, production of SIgA specific for delivered-antigens were detected in intestinal lavage samples from groups immunized via the IM route. This confirmed that live ST variants reached intestinal mucosal immune-sites following systemic immunization and has the ability to activate systemic as well as mucosal immunity.

### The JOL1800 live vaccine vector is DIVA-capable

**Figure 4:**
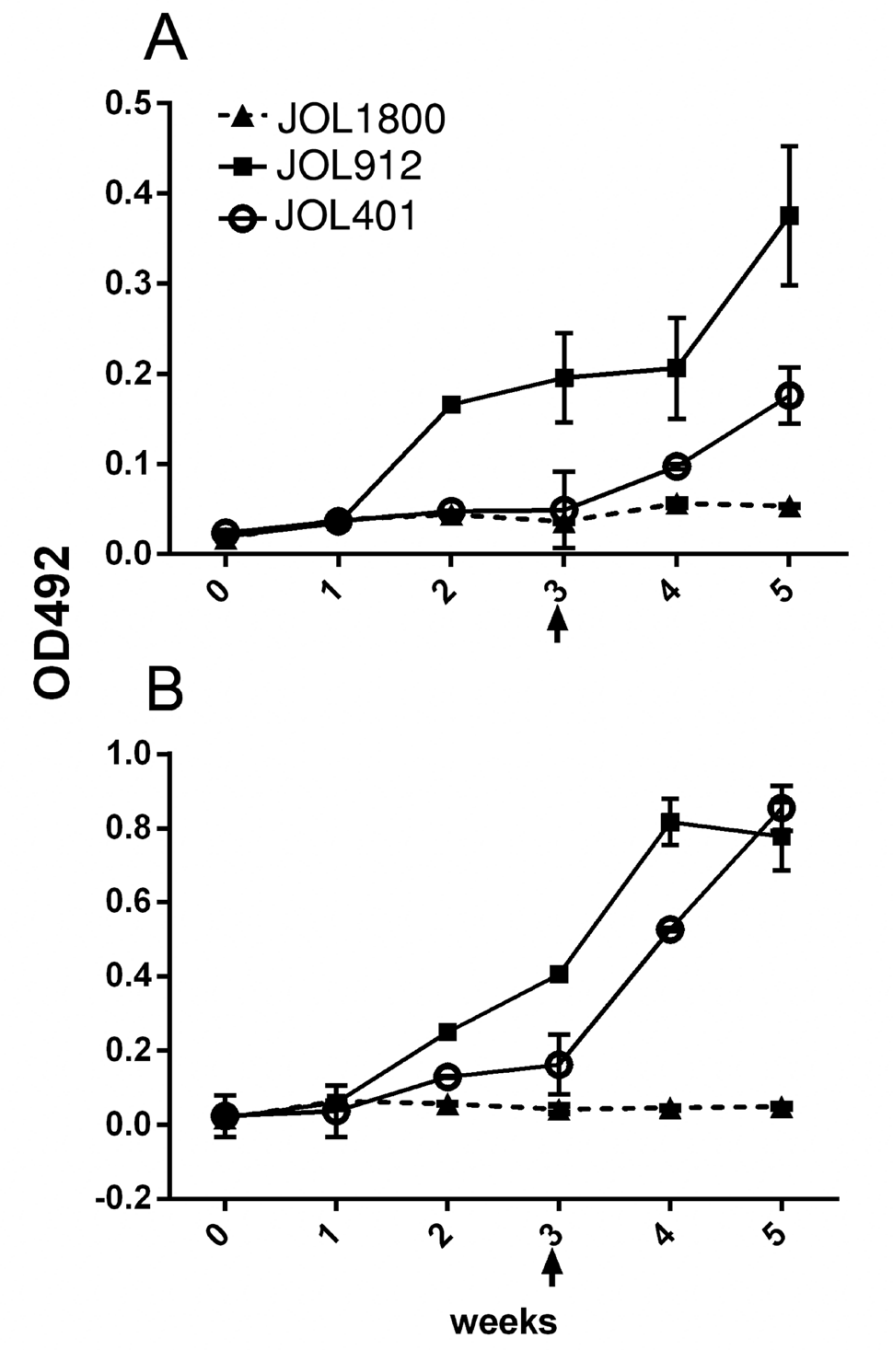
Assessment of DIVA capability Mice were inoculated with the *Salmonella* strains to assess induction of LPS antibodies. The smooth strains JOL401 and JOL912 induced LPS specific antibodies in the inoculated mice while rough JOL1800 did not induced detectable levels. In order to rule out that JOL1800 strain might be extremely attenuated and thus is incapable of inducing any LPS specific bodies, a booster dose at the 3^rd^ week post inoculation was given. However, the booster dose did not further elicit the production of anti-LPS antibodies which revealed the stringency of DIVA potential of the JOL1800 strain. (**A**) Anti-LPS IgG responses induced by oral immunization of *Salmonella* vector strains. (**B**) Anti-LPS IgG responses induced by IM immunization. Arrow depicts time point of booster immunization.

To validate the DIVA potential of the JOL1800 delivery system, reactivity of inoculated mice serum IgG with purified ST LPS was tested using indirect ELISA format. Relative OD_492_ levels were determined between the sera of JOL401, JOL912, and JOL1800 inoculated mice (Figure 4). The smooth strains JOL401 and JOL912 induced LPS specific antibodies in the inoculated mice, while JOL1800 did not induce detectable levels. To further rule out that the JOL1800 strain could be extremely attenuated and thus incapable of inducing any LPS specific bodies, a booster dose was given 3 weeks post inoculation. The booster dose did not further enhance the production of anti-LPS antibodies in JOL1800 immunized mice, which confirmed the DIVA potential of the JOL1800 strain.

### JOL1800-delivered antigens are more highly displayed on antigen-presenting cells

**Figure 5:**
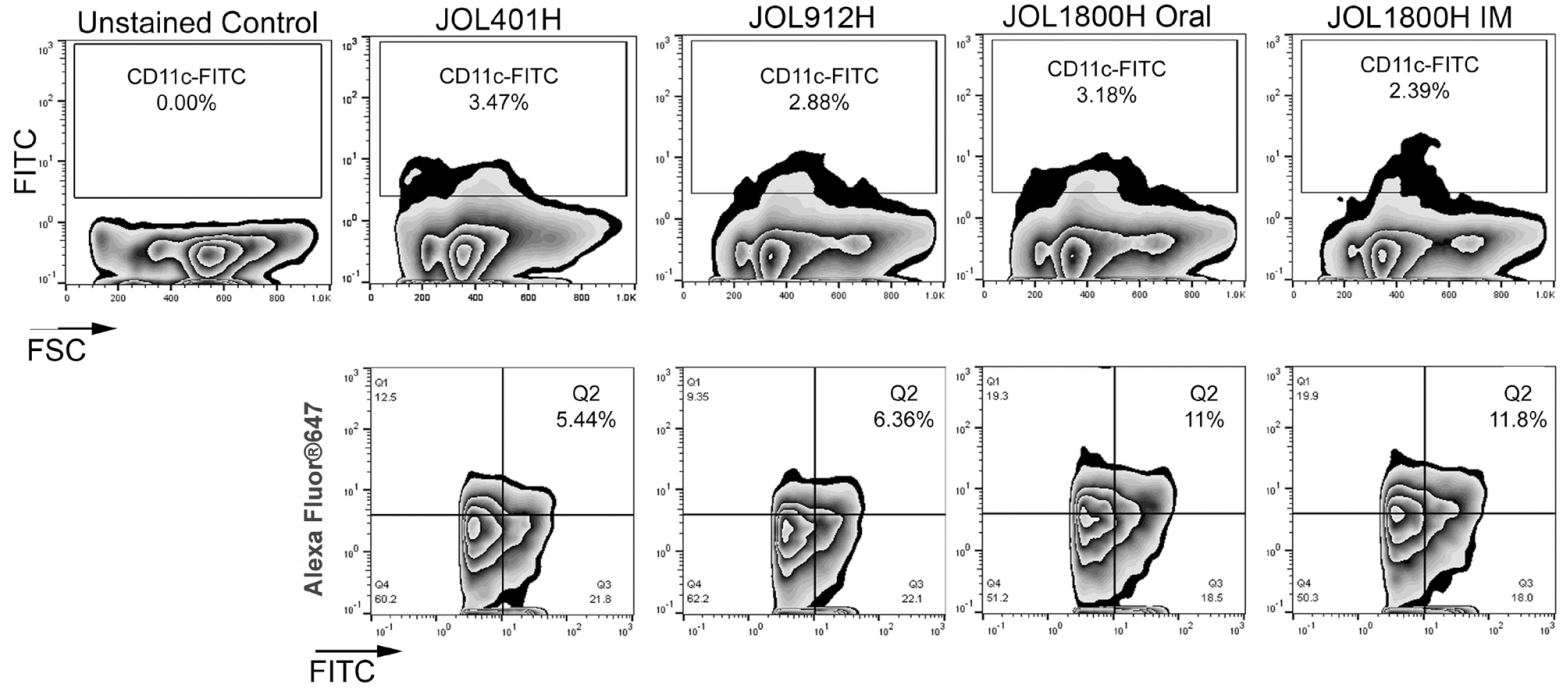
FACS analysis of antigen presentation The fractions of DC presenting delivered antigen was determined on murine splenocytes using flow cytometric analysis. Mice were inoculated with hemagglutinin delivering ST strains, viz. wild type strain (JOL401H), smooth strain (JOL912H) and rough strain (JOL1800H). At 5 days post inoculation, mice were euthanized, and splenocytes were harvested aseptically and subjected to FACS analysis. DC population was gated with anti-CD11c-FITC and the delivered antigen HA protein was detected with rabbit anti-HA primary antibody and anti-rabbit IgG-AlexaFluor647.

To investigate and quantify *in vivo* DC uptake and antigen presentation elicitation of the JOL1800 delivery system, mice were inoculated via oral or IM with JOL401H, JOL912H, and JOL1800H ST strains expressing and delivering the HA antigen. Mice were euthanized, and splenocytes were harvested. The fraction of dendritic cells (DCs) presenting delivered antigen was determined on total murine splenocytes using flow cytometric analysis (FACS). The DC population was gated with anti-CD11c-FITC, and the delivered antigen HA protein was detected using rabbit anti-HA primary antibody and anti-rabbit IgG-AlexaFluor647 (Figure 5). The frequency of DC displaying HA was 6.22% ±2.36, 6.63% ±1.65, 10.36% ± 1.23, and 12.32% ± 3.19 for JOL401H, JOL912H, JOL1800H Oral, and JOL1800H IM, respectively. Significant frequency difference (*p* ≤ 0.05) was observed between the smooth and rough type strains.

**Figure 6:**
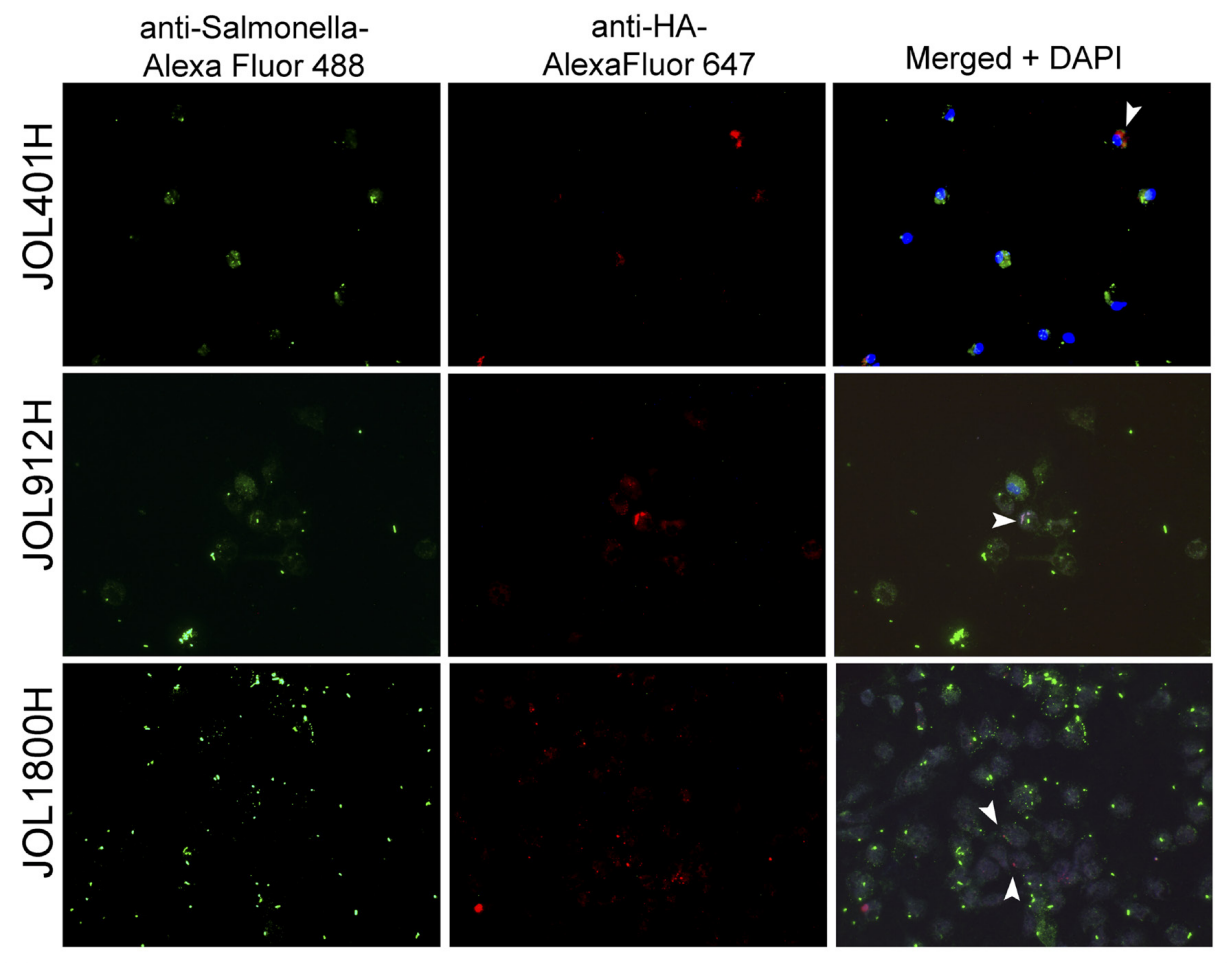
Fluorescence microscopic observed of delivered antigen display on mock infected macrophages Murine intraperitoneal macrophages were infected *in vitro* at 10:1 MOI with hemagglutinin delivering ST strains, viz. wild type strain (JOL401H), smooth strain (JOL912H) and rough strain (JOL1800H). At 16 hr post infection, the cells were fixed and subjected to immunofluorescence microscopic analysis. Macrophages infected with JOL1800H showed maximal display of *Salmonella* and HA antigens which was followed by JOL912H. Arrowhead depicts HA antigen bound fluorescence signal (red) on the surface of macrophage; Magnification- 200X.

To visualize the magnitude of antigenic display at the cellular level, murine intraperitoneal macrophages were infected *in vitro* at 10:1 MOI with hemagglutinin delivering ST strains (Figure 6; Supplementary Figure 5). At 16 h post infection, the cells were fixed and subjected to immunofluorescence microscopic analysis. Wild type JOL401H, which was more resistant to lysosomal degradation, was seen almost intact inside the macrophages, and minimal ST antigen or HA antigen was observed. Macrophages infected with JOL1800H showed maximal display of ST and HA antigens, followed by JOL912H. Hence, direct visualization revealed that antigenic display and presentation were increased not only in the cell population, but also in individual cells. Collectively, the FACS data and immunofluorescence microscopic analysis confirmed enhancement of antigenic display of the rough ST vector and the delivered antigen by antigen-presenting cells.

### Pre-existing wild type ST-immunity has minimal effect on the efficacy of JOL1800 antigen delivery

**Figure 7:**
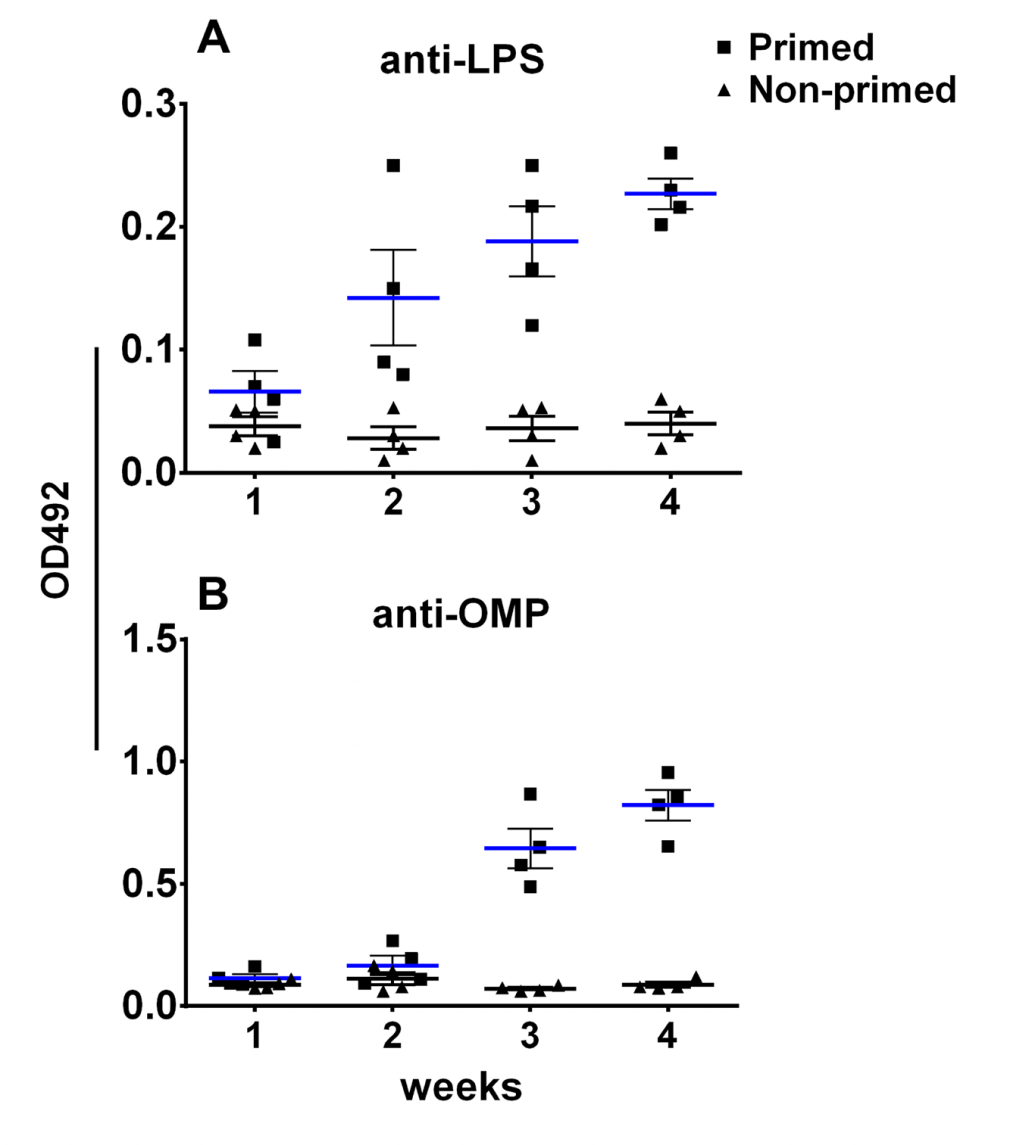
Immune priming of mice with live *Salmonella* The effect of priming with *Salmonella* on the humoral immune response following oral inoculation was monitored. (**A**) Production of anti-LPS IgG in primed versus non-primed mice. (**B**) Production of anti-OMP IgG in primed versus non-primed mice. Blue bar represents mean titre for primed mice; black bar represents mean titre for non-primed mice.

**Figure 8:**
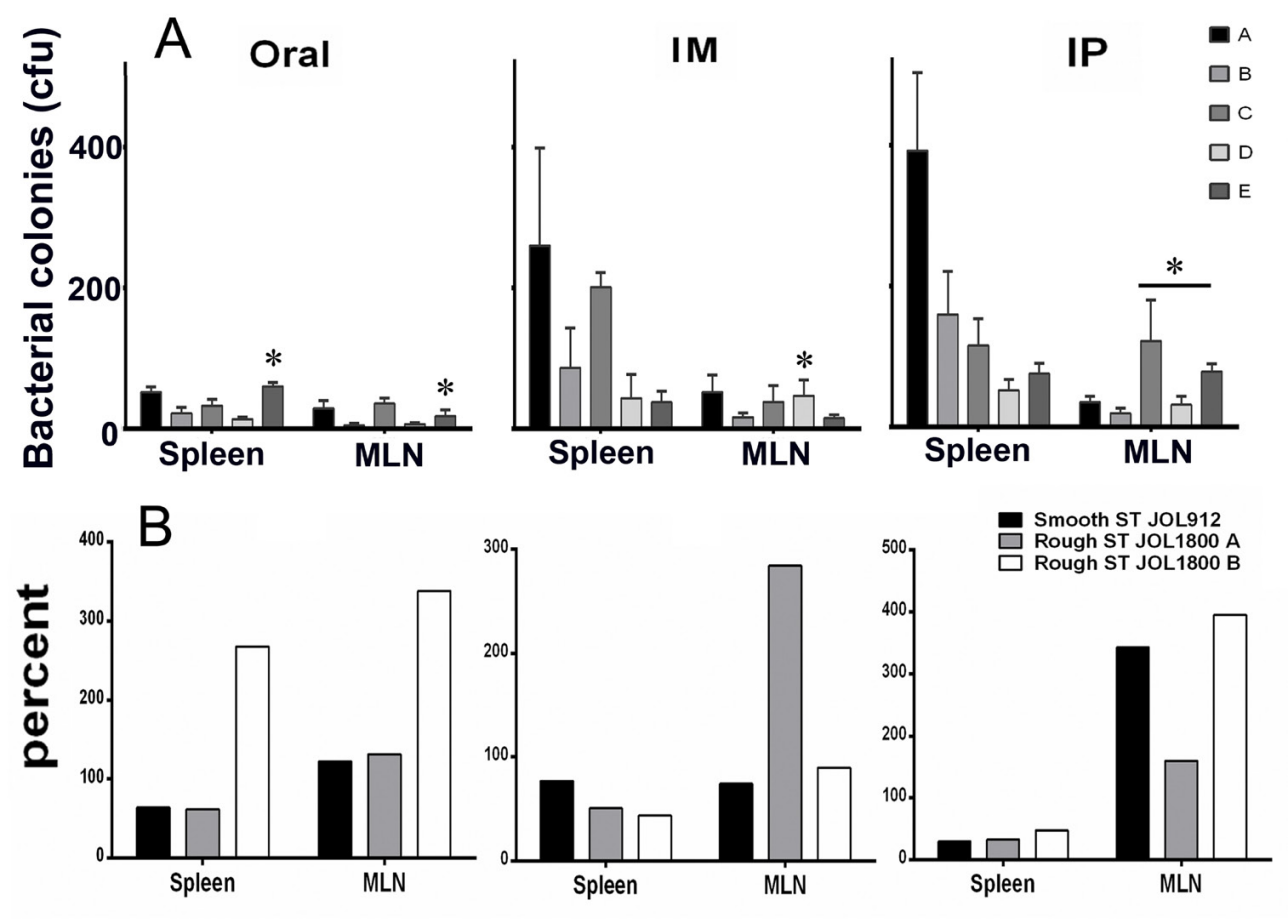
Effects of priming on colonization of rough and smooth *Salmonella* The fate of vector vaccine inside host harboring pre-existing *Salmonella* immunity was investigated. To simulate natural infection, mice were inoculated orally with smooth wild type ST strain. Effects of priming on colonization of vector in spleen and mesenteric lymph nodes (MLN). (**A**) Bacterial recovery of *Salmonella* vector strains from primed and non-primed mice. Group A and B- non primed, group C-E- primed; group A and C- inoculated with smooth JOL912 (2 × 10^6^ cfu), group B and D- inoculated with rough JOL1800 (2 × 10^6^ cfu); group E- inoculated with rough JOL1800 (1 × 10^8^ cfu). ^*^indicates significant difference (*P* ≤ 0.05) as compared to corresponding non-primed controls group A or B. (**B**) Percent fraction of vector strain recovered from primed mice. The percentage was calculated as- (mean vector CFU recovered from primed mice divided by mean vector CFU recovered from un-primed mice) × 100. ST- *Salmonella Typhimurium*; JOL1800 A- 2 × 10^6^ cfu vector dose; JOL1800 B- 2 × 10^8^ cfu vector dose.

**Figure 9:**
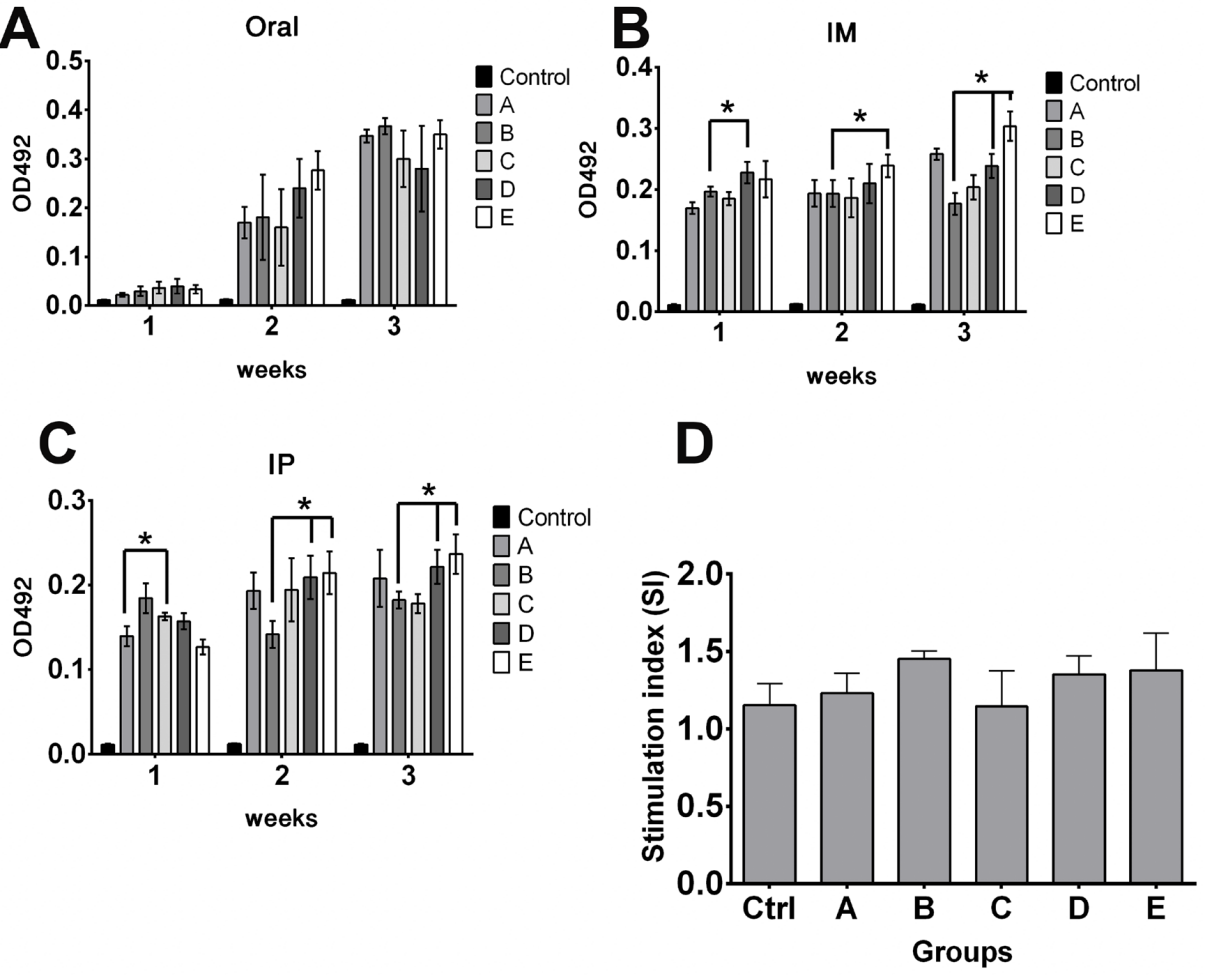
Effects of priming on antigen delivery The fate of delivered antigen (*Brucella* lumazine synthase) inside host harboring pre-existing *Salmonella* immunity was investigated. To simulate natural infection, mice were inoculated with smooth wild type ST strain. Effects of priming on humoral and cellular immune responses of delivered antigen in primed and non-primed mice were determined. (**A**) Effects of priming on IgG responses following oral delivery. (**B**) Effects of priming on IgG responses following IM delivery. (**C**) Effects of priming on IgG responses following IP delivery. Control group represents naive mice anti-BLS IgG levels. (**D**) Effects of priming on splenocytes proliferation responses following IM delivery. Cellular immune response against delivered *Brucella* lumazine synthase (BLS) antigen was not decreased. ^*^indicates significant difference (*P* ≤ 0.05) as compared to corresponding non-primed controls group A or B. Group A and B- non primed, group C-E- primed; group A and C- inoculated with smooth JOL912 (2 × 10^6^ cfu), group B and D- inoculated with rough JOL1800 (2 × 10^6^ cfu); group E- inoculated with rough JOL1800 (1 × 10^8^ cfu).

To assess the effect of pre-existing antibodies on the bacterial vector and to validate whether removal of LPS could minimize vector colonization and efficacy, mice were primed with wild type ST via oral inoculation. Vector strains were used to inoculate JOL401 wild type-primed and control non-primed mice at 4 weeks post priming, when anti-LPS antibodies were at plateau. Progression of the humoral response against the smooth ST was monitored to ascertain the levels of priming induced following immunization (Figure 7). At 9 days post vector-delivery, with the establishment of infection, non-primed naive mice showed a surge in ST vector cfu (Figure 8). However, in primed mice, reduction in the cfu of smooth and rough strains vectors was observed. Further, the rough strain JOL1800 showed lower bacterial recovery, which in part may be due to the attenuation profile. However, the percent reduction in colony count was greater in the JOL912 strain. Further, a higher dose overcomes this pre-existing immune response effect, and the amount of bacteria recovered from spleens was adequate for effective antigen delivery. Further, to investigate the effects of priming on humoral and cell-mediated responses of the delivered antigens, IgG levels and lymphoproliferative index were determined on mice immunized with the JOL-delivery strains. Our data revealed that priming did not affect the humoral response against the BLS antigen delivered via rough JOL1800 (Figure 9). Further, the higher dosage of JOL1800 revealed the highest relative antibody levels. Between oral, IM, and IP routes, antigen delivered via IM showed the strongest humoral response to the delivered antigen. In this study, we also investigated the outcome of cellular immunity induced in primed and non-primed hosts. Our data revealed that the cellular immune response against delivered *Brucella* lumazine synthase (BLS) antigen was not affected, at least with the current experimental setup (Figure 9D). Hence, we deduced that antigen can be effectively delivered via the rough JOL1800 strain, even in a host immune-primed with natural levels of wild type smooth ST infections.

## DISCUSSION

The present study reported the generation of a suitable bacterial vector for the use in vaccine antigen delivery. However, there are certain area that needed to be address, this includes but not limited to, vector-dose optimization, inoculation route optimization, post vector-delivery protection assays, studies involving delivery of therapeutic molecules, and studies involving immune-compromise host.

Lipopolysaccharide represents the major surface antigen of gram-negative bacteria and harbors binding-sites for antibodies [37]. LPS is important in the recognition and elimination of bacteria by the host immune system [34, 38]. In this study, the rough ST delivery strain JOL1800 was generated from a parental *lon*-*cpxR*-*asd* mutant ST strain via *rfaL* gene deletion. The *rfaL* gene (synonyms: *rfbT*, *waaL*) encodes for an O-antigen ligase protein involved in the LPS core biosynthesis pathway, which is part of bacterial outer membrane biogenesis [39]. Loss of *rfaL* resulted in loss of all O-Ag side-chains (Figure 1B), while the core oligosaccharide remained intact [40]. In our previous studies, we reported that *lon-cpxR* mutant *Salmonella*e resulted in increased bacterial capsular polysaccharide production [19, 41], an increase in macrophage-bacterial uptake [42], and attenuation as indicated by an oral-lethal dose 50 (LD_50_) of 4.1×10^9^ cfu [41] and greater than 2.8 × 10^10^ cfu [42] in BALB/c mice and brown nick chickens, respectively. While the degree of attenuation expressed in terms of LD_50_ is important for determining the upper dosage limit of the vaccine-vector, studies measuring residual virulence for the purpose of antigen delivery may be more meaningful. Recovery time and residual virulence parameters express the time duration and quantity of residual bacteria present in the host system, respectively, and they represent the availability of vector for antigen production/delivery inside the host. Based on the present data, the RT_50_ of JOL1800 was determined to be around 5 days in both oral and IM routes. In summation, JOL1800 is highly attenuated and thus facilitates higher vector-dosing administration, and the duration and residual quantity of colonization were suitable for effective antigen delivery, as suggested by humoral and cell-mediated immune responses against the delivered antigens.

We affirmed in this report that live ST vectors can remotely activate mucosal immunity via systemic immunization, and vice versa. It was evident that live ST secretes antigen payloads at immunological active sites other than the site of immunization. This phenomenon added the advantage of using a live ST vector system, as live *Salmonella* bacteria may migrate to the intestinal mucosal sites post intramuscular inoculation [19]. Remote activation of the mucosal immune response is a recognized phenomenon that is also the basis of the common mucosal immune response [43, 44]. However, remote mucosal immune-activation in most cases is achievable only via mucosal immunization routes such as, oral, nasal, rectal, or vaginal [44]. Moreover, the common mucosal immune response is rather compartmentalized and localized [43]. We validate that IM immunization of LA and HA delivered by ST in mice could generate strong anti-LA and HA SIgA antibodies in the intestines (Figure 3), which is induced by ST operating in the MLNs of mice from where the organism is readily isolated (Figure 8).

In the present study, antigen display/presentation was determined at 5 days post-inoculation, as the number of ST present in the spleen was comparable among the inoculation mice, and the majority of the mice were ST*-*positive (Table 2). Based on our delivered-antigen display experiment, the number of bacteria recovered from spleens does not directly represent the amount of bacterial/bacterial-delivered antigens processed or presented by APC. Mice inoculated with the JOL401 strain exhibited the highest bacterial recovery at each time point; however, the FACS analysis did not reflect higher antigen signals from the gated CD11c^+^ DCs from the pool of splenocytes. In contrast, with comparative lower splenic bacterial recovery levels, the frequency of HA-positive CD11c^+^ DCs was elevated in the group inoculated with JOL1800 (Figure 5). The exact mechanism explaining how processing and thereby antigen display were higher in rough strain delivery is unclear; however, it may be related to inhibition of lysosomal degradation by the wild type strain [45]. While the wild type JOL401 strain evaded lytic killing and continued replication inside the ST*-*containing vacuoles, the deficiency of Lon protease repression in JOL912 and JOL1800 Δ*lon* mutants may have resulted in the induction of apoptosis [46], thereby releasing bacteria from their replicating niche. It was reported that LPS is not involved in the inhibition of phagolysosomal degradation by phagocytic cells [45], and it was reported that *Salmonella* strains induce delayed apoptosis dependent on SPI-2 function that requires LPS for induction [47]. Moreover, LPS is involved in the activation of lysosome-associated membrane proteins (LAMP), particularly LAMP-3, which is important for *Salmonella* intracellular proliferation [48]. Hence, the absence of LPS could result in decreased activation of LAMP-3 and also decrease intracellular proliferation.

Wild type ST are intracellular pathogens that remain restricted to the endosomal compartment of eukaryotic cells, resisting nonspecific killing mechanisms [49]. However, resistance to killing by the smooth type could result in minimal processing of the antigen payload. Hence, the vector component should be readily taken up and processed. One concern with the use of ST strains as a vaccine carrier expressing heterologous antigens is the effect of introduction into immunized hosts or the repeated use of the organism [50]. Thus, whether preexisting immunity interferes with the subsequent use of live ST vaccine vectors may depend on timing and the genetic characteristics and immunogenicity of the strain. With the appropriate vaccine strain, preexisting immunity should not preclude the reuse of carriers or their use in areas where individuals have been previously exposed to *Salmonella*e [50]. In addition, some findings suggest that ST vaccine vectors cannot be employed to deliver multiple doses of a vaccine antigen [51]. It is likely that the replication and spread of the bacteria were curtailed by the presence of residual LPS antibodies and immunological memory due to the first immunization [51]. We anticipated that removal of immunodominant LPS from the vector would minimize immune recognition and bacterial clearance in wild type-primed host. In the present study, mice were primed with wild type ST to mimic natural infection and to induce pre-existing immunity. As it would occur in nature, smooth strain JOL401 was inoculated orally for this purpose. The levels of anti-LPS and anti-OMP antibodies were monitored on a weekly basis using ELISA. Also, BLS-specific lymphoproliferative responses were measured at 21 days post-priming (Figure 9D), and ST vectors were inoculated at 4 weeks post priming. At 9 days post-inoculation, a reduction in all strains due to the immune response was observed in primed mice (Figure 8). The number of bacteria recovered from the spleen was lower for the rough JOL1800 strain as compared to JOL912, this finding is anticipated as JOL1800 is more attenuated than JOL912. However, considering the degree of reduction using non-primed mice as a reference, the smooth strain JOL912 was more affected by priming and prior exposure to the smooth wild type strain. The observed phenomenon may be, in part, due to anti-LPS immune responses in mice.

Taken together, our data support that the JOL1800 rough ST delivery vector is adequately attenuated to permit high-dosage deployment and ideally resides in the host system long enough to achieve the required immune response. We also demonstrated that the rough JOL1800 phenotype assisted in enhanced-antigen presentation and processing of the delivered antigen. Removal of O-Ag from the strain also assisted in avoiding wild type pre-existing immunity. In addition, *Salmonella* serotyping is conventionally performed based on seroreactivity against O-Ag; in primed hosts, the major anti-*Salmonella* antibody is generated against O-Ag, deployment of JOL1800 delivery in the livestock sector will not interfere with LPS-based *Salmonella* Typhimurium diagnostics and serotyping, as JOL1800 is DIVA-enabled. These findings suggest that rough JOL1800 can be used for safe and efficient antigen delivery.

## MATERIALS AND METHODS

### Animals and ethics statement

All animal experimental procedures were approved (CBNU2015-00085) by the Chonbuk National University Animal Ethics Committee in accordance with the guidelines of the Korean Council on Animal Care and Korean Animal Protection Law, 2007; Article 13 (experiments with animals). All animals were purchased from Koatech (Pyeongtaek, Gyeonggi-do, Korea). All female animals were used for the study; two New Zealand white rabbits (8-9 weeks age) and specific pathogen-free BALB/c inbred mice (4-5 weeks average age) were kept under air-conditioned rooms, maintained humanely and were provided water and antibiotic-free food *ad libitum*. Rabbits were housed 1 animal per cage and mice were housed at 5 animals per cage. Animals were monitored twice daily for behavioral and physiological signs. During blood sample collections, rabbits were secured with large cloth to avoid inadvertent movements. After wiping with xylene and alcohol swabs, approximately 4-5ml blood is drawn from the marginal ear vein. At the end of serum complement harvesting, the rabbits were released back to rabbit farm. At the completion or end point of experiments, mice were euthanized by cervical dislocation strictly following standard procedures. All efforts were made to ensure humane handling of animals and to minimize animals suffering.

### Bacterial strains, plasmids, and primers

Bacterial strains used in this study are described in Table 1. The aspartate β-semialdehyde dehydrogenase (*asd*) gene-deleted *Salmonella enterica* serovar Typhimurium (ST) strains were grown at 37° C in lysogeny broth (LB) containing 50 µg/ml diaminopimelic acid. Temperature-sensitive bacterial strains were grown at 28° C in LB broth. For induction of red lambda genes, strains were induced in LB broth containing 0.2% L-arabinose. Bacterial strains were stored as frozen cultures in LB broth containing 20% glycerol at –80° C until use.

### Construction and characterization of a live ST bacterial vector

The knock out mutant strain was constructed per the previously described protocol [52]. Briefly, prior to the target gene deletion from the host strain, JOL912 was electroporated with the helper plasmid pKD46, which provides the inducible red lambda components required for recombination. The target gene *rfaL* was replaced with the *cat*^*R*^ gene contained on a linear PCR product amplified from the pkD3 plasmid. Recombinant clones were selected by plating on LB broth containing 25 µg/ml chloramphenicol. The knockout event was confirmed using outer and inner PCR primers. Finally, the antibiotic resistance cassette was eliminated using the helper plasmid pCP20. The PCR primers used for construction are listed in Table 1.

SDS-PAGE and silver staining demonstrated validation of the rough phenotype. For this purpose, LPS was extracted using a phenol-based commercial LPS extraction kit (iNtRON Biotechnology, South Korea). Purified LPS was electrophoresed on 15% PAGE gels; after fixation, the gel was stained using a silver staining kit (Silver Stain Plus, Bio-Rad, USA). An LPS oxidation step using 200 µL of periodic acid (20% w/v) was included in the staining process.

### *In vitro* complement sensitivity assay

The serum complement sensitivity of ST strains was ascertained with naive rabbit serum, using protocol described earlier [53]. Fresh serum, negative of anti-ST antibody was collected from rabbits and used for the assay. All bacterial strains were grown to late log phase, and 1 × 10^3^ cfu/100 µl each of JOL401, JOL912, and JOL1800 cultures were incubated separately with 100 µL PBS, 100 µL 50% serum complemented, and 100 µL de-complemented serum for 1 h at 37° C. After incubation, each of the test lots was plated onto respective LB agar plates with or without 2,6-diaminopimelic acid supplementation. The data are represented as the mean percent of inoculum = (CFU_treatment_/CFU_PBS_) × 100.

### Recovery of ST strains

A total of 128 mice were divided equally into four groups (*n* = 32). At 4 weeks of age, the mice were inoculated with 1 × 10^7^ cfu of JOL401, JOL912, and JOL1800 strains orally, and the fourth group was inoculated with 1 × 10^7^ cfu of JOL1800 intramuscularly (IM). For the purpose of this experiment, Δ*asd* strains JOL912 and JOL1800 were electro-transformed with the *asd*^*+*^ plasmid pJHL65. At days 3, 7, 14, and 21 post inoculation, eight mice from each group were euthanized by cervical dislocation, and splenic samples were collected. To determine the presence of ST strains in the spleens, the organ was homogenized in 2 mL buffered peptone water (BPW, Becton, MD, USA). One hundred µL homogenate was directly inoculated and spread on brilliant green agar plates (BGA) and incubated overnight at 37° C. In parallel, the remaining BPW samples were further enriched with Rappaport-Vassiliadis (RV) broth by incubation at 37° C for 48 h. The ST-like colonies obtained were finally confirmed by PCR using ST specific primers [54]. The RT_50_ of ST strains was determined using the graphical statistical method described earlier [55–57].

### Humoral immune response against delivered antigens

A total of 80 mice were equally divided into eight groups (*n* = 10). All mice were inoculated at 4 weeks of age with smooth or rough strains delivering *Lawsonia* auto-transporter protein A (LA) or hemagglutinin from H1N1 (HA); JOL912L, JOL912H, JOL1800L, or JOL1800H strains, either orally or IM with 10^7^ and 10^6^ cfu in 100 µL volume, respectively. Naïve un-inoculated mice (*n* = 6) were also maintained to serve as a reference control. Serum and intestinal lavage samples were collected at weekly intervals for 5 weeks post inoculation. Indirect ELISA was conducted as per protocol described previously [25] using purified LA and HA recombinant proteins. Briefly, 500 ng/well of antigens was used for coating ELISA plates (MaxiSorp, NUNC). Primary mouse serum and SIgA samples were diluted at 1:100 and 1:5 with PBS, respectively, and secondary HRP-conjugated anti-mice IgG and IgA antibody were used at concentrations of 1:8000 and 1:5000 dilutions, respectively. Colorimetric changes due to HRP acting on OPD (Sigma-Aldrich, USA) were measured after 5 min at 492 nm (TECAN, Austria). The values for IgG and IgA binding to respective antigens are expressed as the mean OD value ± SE.

### Quantification and direct visualization of infected cells-antigen display of delivered antigens

The fraction of mice CD11c^+^ DCs displaying the delivered antigen after *in vivo* inoculation with ST vector was estimated. A total of 32 mice were divided equally into four groups. At 4 weeks of age, mice were inoculated with 10^7^ cfu/100 µL of JOL401H or JOL912H via IM, or JOL1800H via oral as well as IM. At 5 days post inoculation, mice were euthanized, and splenocytes were harvested aseptically and subjected to FACS analysis. For the detection of DCs and the displayed antigen, 10^6^ splenocytes were stained with anti-mouse CD11c-FITC antibodies (Miltenyi Biotec, Germany). The displayed HA antigen was detected using primary rabbit anti-HA antibody (GenScript, USA) at a 1:1000 dilution; Anti-HA polyclonal antibody was used for the experiment in order to detect diverse range of HA peptides presented on the surface of CD11c+ cells. For secondary antibody, goat anti-rabbit IgG H&L conjugated with Alexa Fluor®647 was used at a 1:2000 dilution (Abcam, UK). Relevant isotype controls, internal controls, and unstained cell controls were kept. The splenocyte populations that were positive for CD11c and HA were enumerated, and the mean percent ± SEM was determined.

The frequency of antigens displayed by individual primary intraperitoneal (IP) macrophages after *in vitro* infection with ST vector was ascertained by direct observation following immunofluorescence staining. Primary IP macrophages were harvested from 4-week-old mice following IP inoculation of 5% bovine serum albumen. Macrophages were harvested aseptically and seeded as 5 × 10^6^ cells on 0.2% gelatin-coated glass cover slips that were housed in 6-well cell culture plates containing RPMI-1640 medium (Gibco, USA). After 24 h of culturing at 37° C in a 5% CO_2_ atmosphere, the macrophages were infected with JOL401H, JOL912H, or JOL1800H ST vectors delivering HA antigen at an MOI of 10:1. Infection was initiated by brief centrifugation, and the culture plate was incubated at 37° C in a 5% CO_2_ atmosphere for 30 min. Non-internalized bacteria were washed with PBS, and the cells were further incubated with antibiotics containing RPMI for 48 h. Cells on the cover-slips were fixed with 2% paraformaldehyde and subjected to immunofluorescence staining. ST bacteria were stained with primary chicken anti-*Salmonella* hyperimmune serum (1:1000) and secondary goat anti-chicken IgY H&L-Alexa Fluor^®^ 488 (Abcam, UK) (1:2000). HA antigen was detected using primary rabbit anti-HA antibody (GenScript, USA) at a 1:1000 dilution and secondary goat anti-rabbit IgG H&L-Alexa Fluor^®^ 647 at a 1:2000 dilution (Abcam, UK). Macrophage nuclei were counterstained with 2-(4-amidinophenyl)-1H -indole-6-carboxamidine (DAPI; Sigma-Aldrich, USA) at a 0.5 µg/mL final concentration. The cover-slips were retrieved from the wells, attached to a micro-slide, and then observed under a fluorescence microscope (AX1 Zeiss, Germany).

### Serum IgG-based DIVA capability assessment

A total of 60 mice were equally divided into six groups (*n* = 10). All mice were inoculated at 4 weeks of age, followed by a second dose 3 weeks post inoculation. Mice were inoculated with JOL401, JOL912, or JOL1800 strains, either orally or IM with 1 × 10^7^ and 1 × 10^6^ cfu in 100 µL volume, respectively. Serum samples were collected from 4 mice at weekly intervals for 5 weeks post inoculation. LPS-indirect ELISA was conducted using purified *Salmonella* Typhimurium LPS (L6511 SIGMA, Sigma-Aldrich Co. LLC, USA). Briefly, 500 ng/well of LPS was used for coating ELISA plates (MaxiSorp, NUNC). Primary mice serum was diluted at 1:100 with PBS, and secondary HRP conjugated anti-mice IgG antibody was used at a 1:8000 dilution. Colorimetric changes due to HRP acting on OPD (Sigma-Aldrich, USA) were measured after 5 min at 492 nm (TECAN, Austria). The values for serum IgG binding to LPS are expressed as the mean OD value ± SE.

### Effects of pre-existing immunity on ST vector colonization and efficacy of antigen delivery

A total of 90 mice were equally divided into five groups (*n* = 18). Group A and Group B were comprised of non-primed mice. At 4 weeks of age, Groups C, D, and E were primed with smooth wild type JOL401 at 10^5^ cfu/mouse via oral inoculation. Serum samples were collected weekly until 4 weeks post priming (*n* = 4) to ascertain humoral responses to priming. Anti-LPS and anti-outer membrane protein IgG responses after oral inoculation was monitored on the serum samples using indirect-ELISA as per protocol described elsewhere [26]. Briefly, 600 ng of Omp/well or 500 ng of purified ST- LPS (L6511 SIGMA, Sigma-Aldrich Co. LLC, USA) /well was used to coat ELISA plates (Maxisorp, NUNC). Primary mouse sera were diluted with PBS at 1:100 dilutions. Secondary horseradish peroxidase (HRP)-conjugated anti-mice IgG antibodies were used at 1:5000 dilutions. Colorimetric changes resulting from the action of HRP on OPD (Sigma-Aldrich, US) were measured (TECAN, Austria) at 492 nm, 10 post-development. The values for binding of IgG to respective antigens was expressed as the mean OD value ± standard error of mean (SEM). Cellular immunity induced by priming was also ascertained 4 weeks post priming (*n* = 4). To deliver *Brucella* lumazine synthase (BLS) via JOL strains, the mice groups were inoculated with 2 × 10^6^ cfu of JOL912B (Groups A and C), JOL1800B (Groups B and D), and Group E mice were inoculated with 1 × 10^8^ cfu JOL1800B at 5 weeks post priming via oral, IM, and IP routes. The degrees of splenic and mesenteric lymph node (MLN) colonization and clearance in non-primed and primed mice were ascertained at 9 days post-delivery inoculation. To differentiate wild type and vector strains, chloramphenicol resistance plasmid was introduced in vector strains and selected accordingly. Bacterial recovery and enumeration were performed per the protocol described above. The percentage of cfu recovered from primed mice as compared to non-primed mice was ascertained. Serum samples were collected at weekly intervals for 3 weeks post-delivery (*n* = 6). Outer membrane (OMP)-, LPS-, and BLS-indirect ELISA were conducted using purified *Salmonella* Typhimurium OMP antigen, LPS (L6511 SIGMA, Sigma-Aldrich Co. LLC, USA), and recombinant BLS protein, respectively, on collected serum samples. Lymphoproliferative assays against purified recombinant BLS protein were conducted in the splenocytes of mice pre and post priming (*n* = 6).

### Statistical analysis

Statistical analyses were performed wherever applicable. One-way analysis of variance (ANOVA) and Student’s *t*-tests were used to determine statistically significant differences, with a *P* value ≤ 0.05 considered significant. Tukey’s test was applied for post hoc analysis. Analyses were performed using SPSS 16.0 (SPSS Inc., Chicago, IL, USA).

## SUPPLEMENTARY MATERIALS FIGURES


