# Supplementary material for: Engineering of a rough auxotrophic mutant Salmonella Typhimurium for effective delivery
Source: Oncotarget. 2018 May 22;9(39):25441–57. doi: 10.18632/oncotarget.25192 (PMC5986645; doi:10.18632/oncotarget.25192)
Supplement: Supplementary file 1 [file oncotarget-09-25441-s001.pdf]

Engineering of a rough auxotrophic mutant *Salmonella* Typhimurium for effective delivery

SUPPLEMENTARY MATERIALS

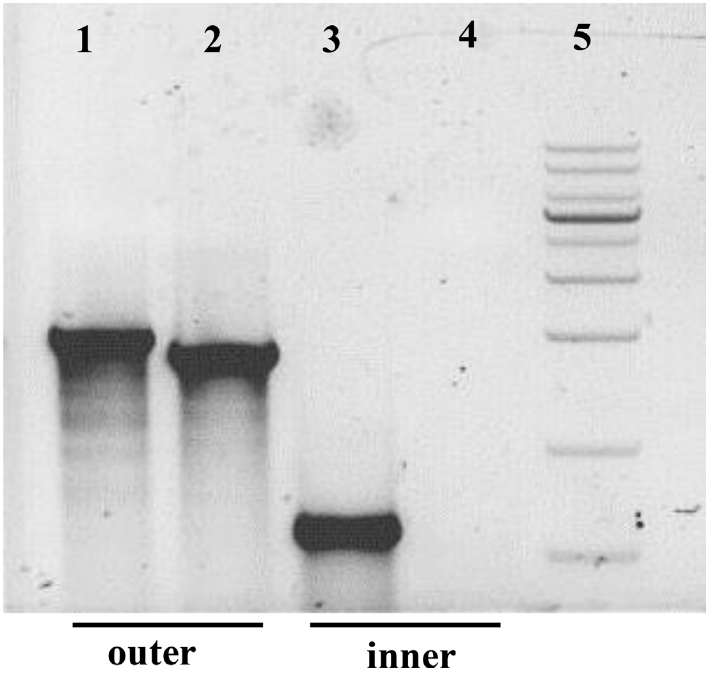

Supplementary Figure 1: PCR amplification of rfaL gene regions, 1% agarose gel image of deletion confirmation. Lane 1, wild type JOL401 DNA band (~2 kbp) amplified using rfaL outer flanking PCR primers; lane 2, mutant JOL1800 DNA band (~1.7 kbp) amplified using rfaL outer flanking PCR primers; lane 3, wild type JOL401 DNA band (~700 bp) amplified using rfaL inner PCR primers; lane 4, absence of amplified band in JOL1800 due to deletion of *rfaL* gene; and lane 5, 1kbp DNA ladder marker.

A

B

C

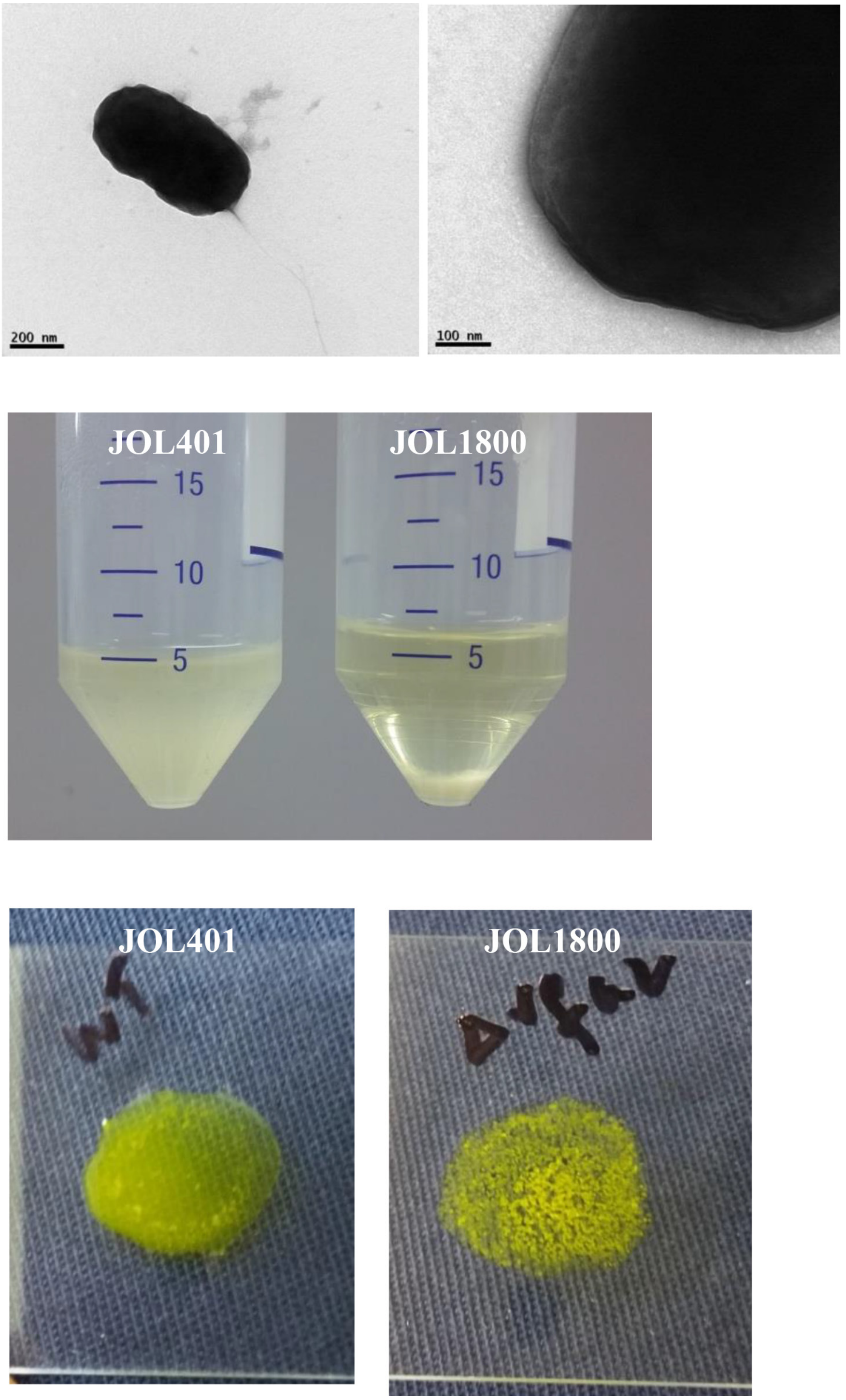

Supplementary Figure 2: Phenotypic characterization of JOL1800. (**A**) Electron microscopy. To ascertain the bacterial morphology and surface integrity, JOL1800 was subjected to electron microscopic analysis. Despite multiple genes affecting membrane synthesis (i.e., *asd, rfaL* genes) deletion, disruption in flagella-assembly, weakened or disruption in membrane structures due to deletion was not evident from direct EM observation. (**B**) Sedimentation Rate. Auto-agglutination of rough strains resulted in heavier bacterial-mass formation, increase sediment rate was observed on JOL1800 cultures. Figure depicts culture settling for ~10 hr. (**C**) Acriflavine agglutination assay. Ten µl of acriflavine solution (0.1%w/v) was mixed with a loop-full of fresh *Salmonella* colony. Agglutination due to hydrophobic interaction validates the surface properties of rough strain. JOL1800 should strong agglutination as compared to wild type JOL401.

j	i	Tij	Nij	Cij	C*ij	CAij	Iij	IAij	NAij	Pij
JOL401	1	3	8	0	0.25	0.25	7.75	27.5	27.75	0.9009
	2	7	8	0	0.25	0.5	7.75	19.75	20.25	2.46914
	3	14	8	1	1	1.5	7	12	13.5	11.1111
	4	21	8	3	3	4.5	5	5	9.5	47.3684
JOL912	1	3	8	0	0.25	0.25	7.75	22.75	23	1.08696
	2	7	8	1	1	1.25	7	15	16.25	7.69231
	3	14	8	2	2	3.25	6	8	11.25	28.8889
	4	21	8	6	6	9.25	2	2	11.25	82.2222
JOL1800ORAL	1	3	8	0	0.25	0.25	7.75	18	18.25	1.36986
	2	7	8	2	2	2.25	6	10.25	12.5	18
	3	14	8	4	4	6.25	4	4.25	10.5	59.5238
	4	21	8	8	7.75	14	0.25	0.25	14.25	98.2456
JOL1800IM	1	3	8	0	0.25	0.25	7.75	18	18.25	1.36986
	2	7	8	1	1	1.25	7	10.25	11.5	10.8696
	3	14	8	5	5	6.25	3	3.25	9.5	65.7895
	4	21	8	8	7.75	14	0.25	0.25	14.25	98.2456

* Observed data modified by Bartlett's cunning
– *j* = (*REF, TEST*), index of the vaccine strain used
– *i* = (1, 2, 3, 4), index of the slaughter time
– $T_{i,j}$ = (3, 6, 9, 12), corresponding slaughter time point in weeks post-inoculation of mice inoculated with strain *j*
– $N_{i,j}$ = number of mice inoculated with strain *j* and slaughtered at each time index *i*
– $C_{i,j}$ = number of mice inoculated with strain *j* found to be 'cured' at slaughter time index *i*
– $CA_{i,j}$ = number of accumulated cured mice at slaughter time index *i* for strain *j*
– $I_{i,j}$ = number of mice inoculated with strain *j* found to be 'infected' at slaughter time index *i*
– $IA_{i,j}$ = number of accumulated infected mice at slaughter time index *i* for strain *j*
– $NA_{i,j}$ = total number of accumulated mice at slaughter time index *i* for strain *j*
– $P_{i,j}$ = probability that a mouse inoculated with strain *j* and slaughtered at time index *i* be cured

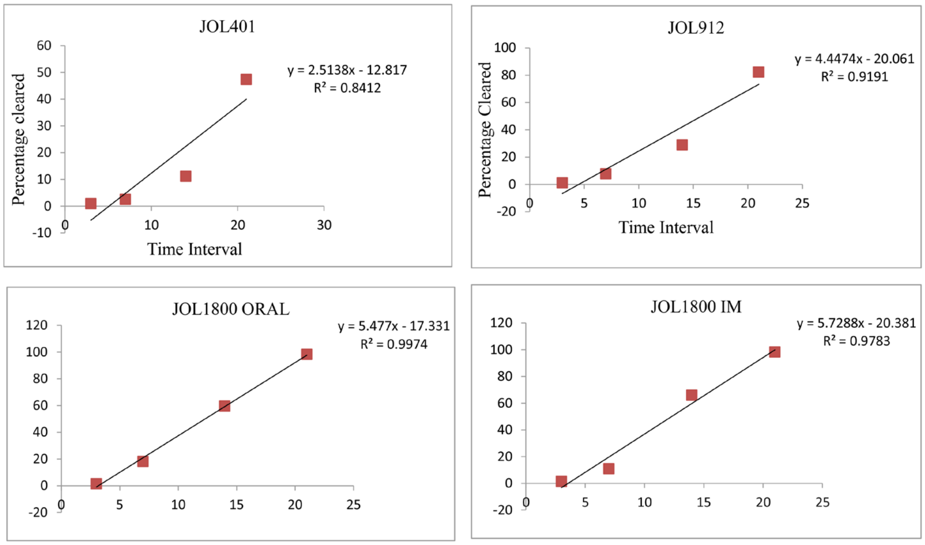

Reference: Pouillot, R., Grilló, M. J., Alabart, J. L., Garin Bastuji, B. & Blasco, J. M. Statistical procedures for calculating the residual virulence of Brucella abortus strain 19 (S19) and Brucella melitensis strain Rev 1 vaccines in mice: theoretical basis and practical applications. *Rev. Sci. Tech.* **22,** 1051–1063 (2003).

Strains	Recovery time (days)		
	**RT16**	**RT50**	**RT84**
**JOL401**	11.5	25.0	38.5
**JOL912**	8.1	15.8	23.4
**JOL1800 ORAL**	-0.2	6.0	12.2
**JOL1800 IM**	-0.8	5.2	11.1

Supplementary Figure 3: Statistical analysis of recovery time (RT) of JOL strains.

A 3 days post-infection

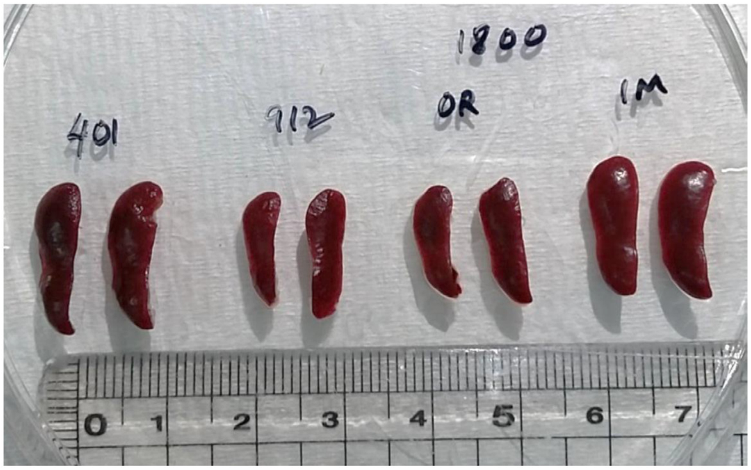

B 14 days post-infection

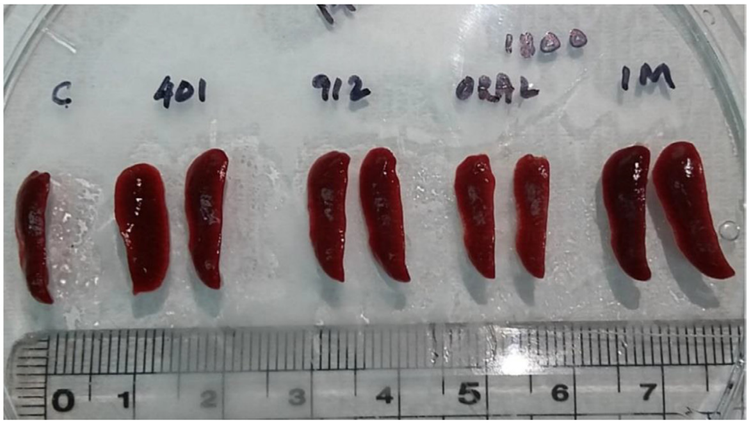

Supplementary Figure 4: Relative splenic morphology and size, post Salmonella strains mock infections, representative spleens isolated from mock infected mice. Splenomegaly and congestion was observed in spleens of JOL401 orally infected mice, splenomegaly was also observed in spleens of mice JOL1800 infected via IM at 3 and 14 days post-infection (**A** and **B**).

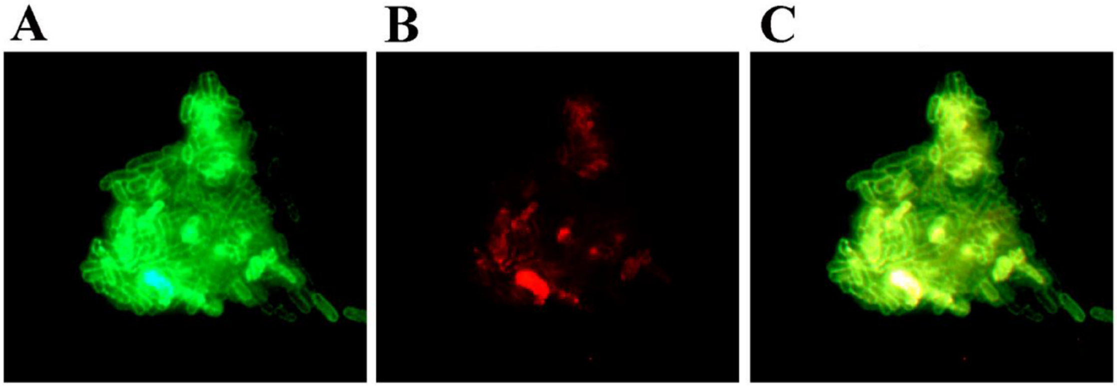

Supplementary Figure 5: Fluorescence staining and microscopy of *Salmonella* expressing HA antigen. (**A**) FITC channel, *Salmonella* bacteria were stained with primary chicken anti-*Salmonella* hyperimmune serum (1:1000) and secondary goat anti-chicken IgY H&L-Alexa Fluor® 488 (Abcam, UK) (1:2000), (**B**) Rhodamine channel, HA antigen was detected using primary rabbit anti-HA antibody (GenScript, US) at 1:1000 dilution and secondary goat anti-rabbit IgG H&L-Alexa Fluor® 647 at 1:2000 dilution (Abcam, UK), (**C**) Merged image. Fluorescence images from green and red channels were merged to form composite image.
